# Efficacy of Wearable Exoskeleton for Gait Recovery in Patients With Stroke: A Multicenter Randomized Controlled Trial

**DOI:** 10.1161/STROKEAHA.125.052763

**Published:** 2025-12-22

**Authors:** Won Hyuk Chang, Tae-Woo Kim, Hyoung Seop Kim, Fazah Akhtar Hanapiah, Jong Weon Lee, Seung-Hyeon Han, Chai Wen Jia, Dae Hyun Kim, Deog Young Kim

**Affiliations:** 1 Department of Physical and Rehabilitation Medicine, Center for Prevention and Rehabilitation, Heart Vascular and Stroke Institute, Samsung Medical Center, Sungkyunkwan University School of Medicine, Seoul, Republic of Korea (W.H.C., D.H.K.).; 2 TBI Rehabilitation Center, National Traffic Injury Rehabilitation Hospital, Gyeonggi-do, Republic of Korea (T.-W.K.).; 3 Department of Rehabilitation Medicine, Pohang Stroke and Spine Hospital, Republic of Korea (H.S.K.).; 4 Faculty of Medicine, Universiti Teknologi MARA, Selangor, Malaysia (F.A.H., C.W.J.).; 5 Department and Research Institute of Rehabilitation Medicine, Yonsei University College of Medicine, Seoul, Republic of Korea (J.W.L., S.-H.H., D.Y.K.).

**Keywords:** exoskeleton device, gait, robotics, stroke, wearable electronic devices

## Abstract

**BACKGROUND:**

Robot-assisted gait training (RAGT) with wearable exoskeletons has the potential to enhance walking in patients with stroke; however, large-scale evidence is inconclusive. The aim of this study was to determine the effect of overground gait training using a torque-assisted exoskeleton in patients with subacute stroke on the recovery of ambulatory function.

**METHODS:**

This international, multicenter, randomized controlled trial enrolled 151 patients with subacute stroke who presented with severe gait impairment but relatively preserved trunk control. Participants were randomized to the RAGT group (30 minutes of conventional gait training plus 30 minutes of exoskeleton) or the control group (60 minutes of conventional gait training), 5 times per week for 4 weeks. The primary outcome was the change in ambulatory function, assessed by the Functional Ambulation Category (FAC) before and immediately after the 4-week intervention. Secondary outcomes included the lower limb strength, balance function. Independent ambulation was reassessed 3 months after the intervention.

**RESULTS:**

A total of 127 participants (56 female (44.1%), mean age, 60.1±13.6 years) completed the 4-week intervention. There were no serious adverse events related to the interventions, and dropout rates tended to be higher in the RAGT group without statistical significance. Both groups showed significant improvement in FAC after the intervention; however, no significant difference between groups (mean change (range), 3.0 (1–5) and 2.5 (1–4) in the RAGT and control group). Both groups exhibited significant gains in lower limb motor function; however, the RAGT group demonstrated a significantly greater improvement in lower limb strength (mean change, 15.9±14.2 and 11.1±11.2 in the RAGT and control group, *P*=0.034).

**CONCLUSIONS:**

Overground gait training using an exoskeleton was not superior to conventional rehabilitation for improving ambulatory function in subacute stroke patients; however, it could provide additional lower extremity motor improvement. These findings suggest that the overground gait training with an exoskeleton might be a potential intervention for patients with subacute stroke.

**REGISTRATION:**

URL: https://www.clinicaltrials.gov; Unique identifier: NCT05157347.

Impaired ambulatory function is a significant contributing factor to poststroke disability. The capacity for independent ambulation has a substantial influence on both physical health and psychological well-being, thereby impacting social participation and overall quality of life.^1^ In the acute stroke phase, ≈80% of patients have ambulatory impairment. Despite the recovery of ambulatory function within the first 18 months after stroke onset, many patients do not fully regain their prestroke mobility.^2,3^ Consequently, gait rehabilitation is imperative for enhancing ambulatory function in patients with stroke.

Robot-assisted gait training (RAGT) has emerged from recognizing the limitations of conventional gait training.^4^ To date, the majority of gait rehabilitation robots used in stroke rehabilitation have been treadmill-based tethered robots designed to facilitate control of the gait cycle.^5^ However, the conditions of treadmill-based tethered robot gait rehabilitation differ from those of actual overground gait.^6^ Therefore, the enhancement in ambulatory capacity subsequent to treadmill-based tethered robot gait rehabilitation may not directly correspond with improved overground gait.^7,8^ Recently, several exoskeletons have been proposed for potential clinical use with the aim of supporting functional ambulation in patients with stroke.^8–11^ Despite the existence of evidence supporting the use of wearable exoskeletons in a small number of studies,^8–11^ a fully powered randomized controlled trial is necessary to elucidate the impact of gait training utilizing a wearable exoskeleton on patients with subacute stroke.

This article reports a randomized controlled trial addressing the hypothesis that being offered overground gait training using a torque-assisted exoskeleton plus conventional gait training is more effective than conventional gait training in patients with subacute stroke on the recovery of ambulatory function.

## Methods

### Data Availability

The data sets used and analyzed in the current study are available from the corresponding author on reasonable request.

### Study Design

This study was an international, multicenter, randomized, controlled trial at 6 sites involving a total of 151 patients with subacute stroke from November 1, 2021 to October 31, 2024. The study was approved by the institutional review board (IRB) of each hospital (IRB of Severance Hospital, South Korea (IRB no. 1-2021-0031), IRB of National Traffic Injury Rehabilitation Hospital (No. NTRH-21016), IRB of Samsung Medical Center (IRB no. 2021-07-021), IRB of the National Health Insurance Service Ilsan Hospital (No. NHIS- 2021-07-029) and IRB of the Universiti Teknologi MARA (No. REC/04/2021 (MR/26)), and conforms to the Declaration of Helsinki. All participants provided written informed consent before starting the study procedures. The detailed rationale and protocol of this study were described in a previous article.^12^ The trial was registered on https://www-clinicaltrials-gov, and this article follows the CONSORT guidelines (Consolidated Standards of Reporting Trials; Supplemental Material).

An independent administrator randomized participants into 1 of the 2 groups using a custom-written script in R version 4.1.3 (R Core Team, 2021: R: A Language and Environment for Statistical Computing. R Foundation for Statistical Computing, Vienna, Austria). A block size of 4 was used, and treatment assignment at the ratio of 1:1 was stratified by each center. After the completion of baseline functional assessments and confirmation of eligibility before the intervention, each participant was randomly assigned to 1 of 2 groups. All outcome measurements and medical record reviews were independently performed by a rater who was blinded to group assignment.

Participants attended a total of 20 sessions, 5 times a week, over 4 weeks, with each session lasting 60 minutes. Functional assessments were conducted before (T0) and immediately after the final intervention (T1). In addition, 3 months after the intervention (T2), all participants were evaluated for gait without physical assistance. This was defined as the ability to ambulate independently for a distance exceeding 10 meters without physical contact, though guidance or monitoring was permitted. This capability was categorized as a Functional Ambulation Category (FAC) score of >3.

### Participants and Recruitment

Patients with stroke admitted to the rehabilitation units of 4 hospitals in Korea (Severance Hospital, Seoul, Korea; TBI Rehabilitation Center, National Traffic Injury Rehabilitation Hospital, Yangpyeong, Korea; Samsung Medical Center, Seoul, Korea; National Health Insurance Service Ilsan Hospital, Goyang, Korea) and 2 hospitals in Malaysia (Daehan Rehabilitation Hospital Putrajaya, Putrajaya, Malaysia and Hospital Al-Sultan Abdullah UiTM) were informed to participate in the study. The attending physician inquired about the patient’s willingness to participate in the study, and the patient decided to participate based on their free will. Inclusion criteria were as follows: (1) adult patients aged ≥19 years, (2) hemiparetic patients after ischemic or hemorrhagic stroke, (3) early subacute stage (from day 7 to <3 months after onset),^13^ (4) severe ambulatory functional impairment with Functional Ambulatory Category (FAC)^14^ score=0 or 1, (5) Trunk Control Test^15^ score ≥ 50, and (6) could walk independently and showed no significant disability (modified Rankin Scale^16^ score ≤1) before stroke onset. Exclusion criteria were as follows: (1) significant difficulty in communication, such as severe cognitive impairment (Mini-Mental State Examination^17^ <10) or severe aphasia, (2) ataxia due to lesion of efferent or afferent pathways of the cerebellum, (3) spasticity of the affected lower extremity (modified Ashworth Scale score ≥2),^18^ (4) severe musculoskeletal disorder of the lower limb, (5) a contracture that limited ambulation, (6) apparent leg length discrepancy of 2 cm or more, (7) a lower limb fracture, open wound, or unhealed ulcer, (8) a severe cardiovascular or pulmonary disease, (9) a history of osteoporotic fracture, (10) another neurological disorder that may affect the ambulatory function (eg, Parkinson disease, multiple sclerosis), and (11) Unwillingness or inability, as determined by the investigator, to follow study procedures.

### Interventions

The RAGT group underwent 30 minutes of conventional gait training and an additional 30 minutes (excluding robot attachment and detachment time) of gait training utilizing an exoskeleton (ANGEL LEGS M20, Angel Robotics, Co, Ltd) in the physiotherapy room. The control group received conventional gait training for the same duration of 60 minutes as the RAGT group in the physiotherapy room. In accordance with the Clinical Practice Guidelines for Stroke Rehabilitation in Korea, certified physiotherapists implemented conventional gait training, including overground walking and balance exercises.^19^ This approach was based on evidence-based medicine and included various rehabilitation techniques tailored to the individual patient’s functional level. The exoskeleton under consideration comprised segmented components designed to provide precise torque assistance at the hip, knee, and ankle joints. The system is equipped with 4 actuators at the hip and knee joints, which are seamlessly integrated with 2 force sensors positioned beneath each ankle-foot orthosis. In the RAGT group, the level of support provided to each participant depended on their functional capacity and ranged from no assistance to active total assistance. During the initial RAGT session, maximum assistance was provided after the exoskeleton was put on. The physiotherapist evaluated the participant’s performance during the intervention and progressively reduced the assistance in each round of RAGT. Before the RAGT, all certified physiotherapists underwent comprehensive training on how to fit and remove the device in an emergency. In addition, an anti-fall harness was provided for use by the physiotherapists during gait training. A detailed description of the intervention was reported in the published study protocol.^12^

### Dropout Criteria

Dropout criteria were as follows: (1) patients who wished to express a desire to discontinue training, (2) patients who did not comply with the guidelines provided by the investigator, (3) patients who required treatment outside the scope of this clinical trial, (4) patients who presented with a serious injury due to an accident such as a fall, (5) patients who attended <80% of the training sessions, and (6) patients who presented with a new major medical condition and therefore require absolute rest for recovery (eg, stroke, myocardial infarction, any other neurological, internal or musculoskeletal condition).

### Outcome Measurements

#### Primary Outcome

The primary outcome was the change in FAC from T0 to T1 to evaluate the recovery of ambulatory function.

#### Secondary Outcomes

Secondary outcomes were the ability to ambulate independently at T2, the changes in functions of the motor function of the affected lower limb, balance, mood, and quality of life from T1 to T2. The Fugl-Meyer Assessment–Lower Extremity^20^ and the lower limb score of Motricity Index^21^ were utilized to assess the motor function of the affected lower limb. The assessment of balance function was conducted by the Trunk Control Test and the Berg Balance Score.^22^ In addition, all participants completed self-administered questionnaires with the Geriatric Depression Scale–short form (GDS-SF)^23^ and EuroQol-5D (EQ-5D)^24^ to assess mood and quality of life.

### Safety Analyses

All participants were monitored for adverse events throughout the intervention period. During each intervention session, medical interviews and physical examinations were conducted to evaluate the presence of musculoskeletal pain and skin lesions. In addition, the severity of spasticity in the affected muscles of the hip extensor, knee extensor, or ankle plantar flexor was evaluated using the modified Ashworth Scale^25^ immediately after the final intervention (T1). The results of these evaluations were meticulously recorded.

### Statistical Analysis

The sample size was calculated based on data from the recent article^26^ to assess the change of FAC from T0 to T1 as the primary outcome in this study, which used a similar study design. It was estimated that a sample size of 150 participants would be necessary to detect a statistically significant difference in the primary outcome with a statistical power of 95% based on the effect size *d*=0.961 at *α*=0.05 with a 25% dropout rate. In the independent ambulation of the present study, participants were evaluated up to T1, and those who could not be assessed at T2 were analyzed using the intention-to-treat analysis with the Last Observation Carried Forward method, replacing outcomes at T1.

The primary outcome with the change of FAC from T0 to T1 did not follow a normal distribution in the Shapiro-Wilk normality test (*P*<0.05). Consequently, a nonparametric test was performed. Furthermore, given the observed discrepancy in FAC scores between the 2 groups at T0, a rank analysis of 1-way ANOVA (ANCOVA) was conducted, with FAC scores at T0 serving as a covariate.

The distribution of all continuous outcomes except the change of FAC was found to be normal (*P*>0.05, according to the Shapiro-Wilk normality test). To make a comparison between the demographic and functional characteristics of the 2 groups, an independent *t* test was used for continuous variables. A paired *t* test was used to assess the functional scores between T0 and T1 within the group analysis. *χ*^2^ analysis was used for categorical variables. The statistical analyses were conducted using SPSS version 23.0 (SPSS, Chicago, IL). The term statistical significance was defined as a *P* value of <0.05.

## Results

### Participant Characteristics

**Figure 1.**
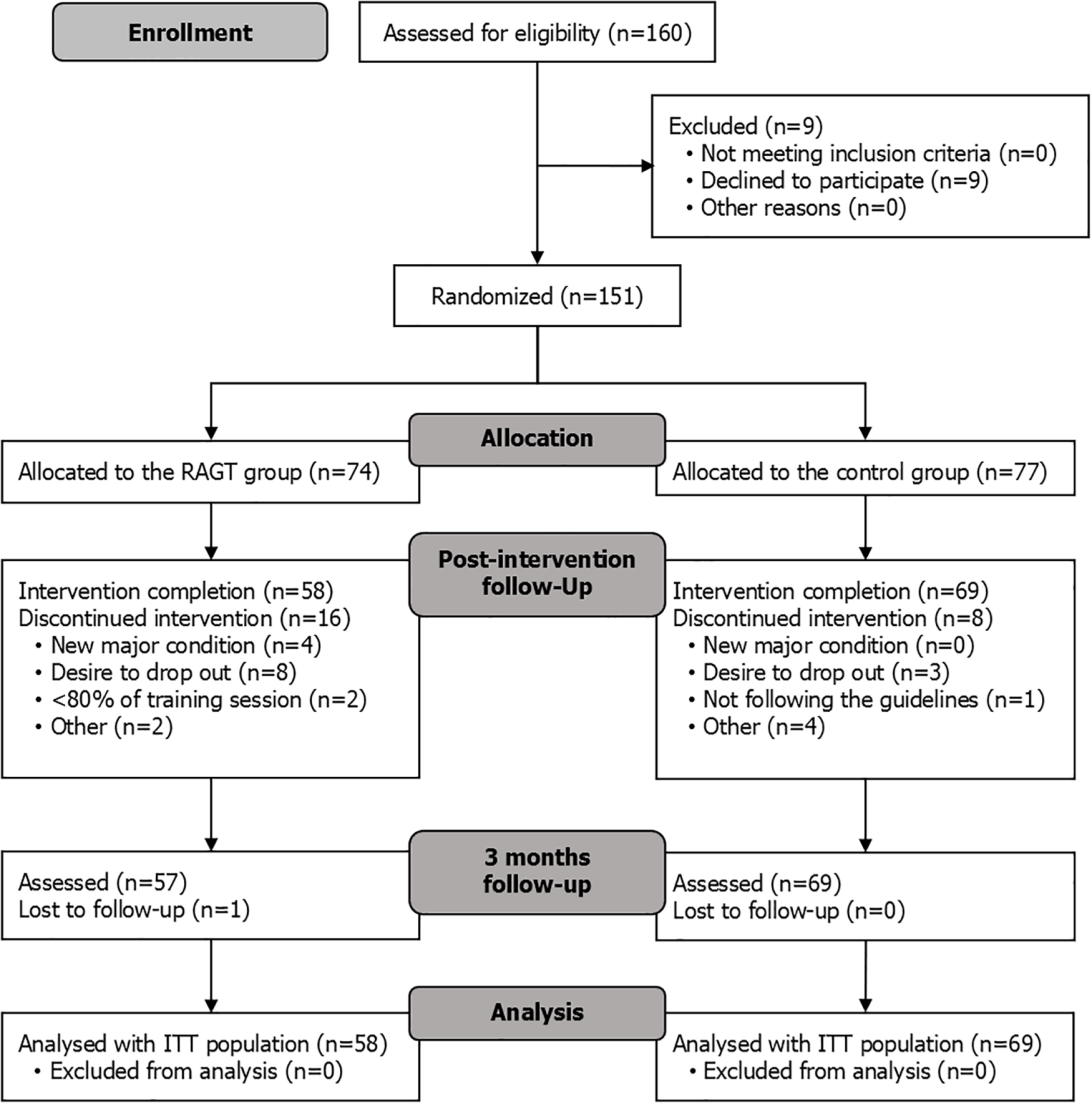
**CONSORT (Consolidated Standards of Reporting Trials) flow diagram of the study.** ITT indicates intention-to-treat; and RAGT, robot-assisted gait training.

A total of 151 participants were randomly assigned to the 2 groups (74 in the RAGT group and 77 in the control group). A total of 127 participants completed the 4-week intervention. At T2, only 1 participant in the RAGT group was not available for further observation. Finally, the data of a total of 127 participants (58 in the RAGT group and 69 in the control group) were included in the analysis (Figure 1). The dropout rate in the RAGT group tended to be higher than in the control group without statistical significance (*P*=0.075). During the intervention, 16 participants from the RAGT group withdrew due to new major illness unrelated to the intervention (n=4), desire to stop (n=8), attending <80% of sessions (n=2), and other personal reasons (n=2). In the control group, 8 participants withdrew, citing a desire to stop (n=3), not following the guidelines (n=1), and other personal reasons (n=4).

Table 1 presents the baseline characteristics of the RAGT and control groups. There was no significant difference in the demographic and clinical characteristics between the RAGT and control groups. However, FAC at T0 in the RAGT was considerably lower than that in the control group (*P*<0.05).

**Table 1.**
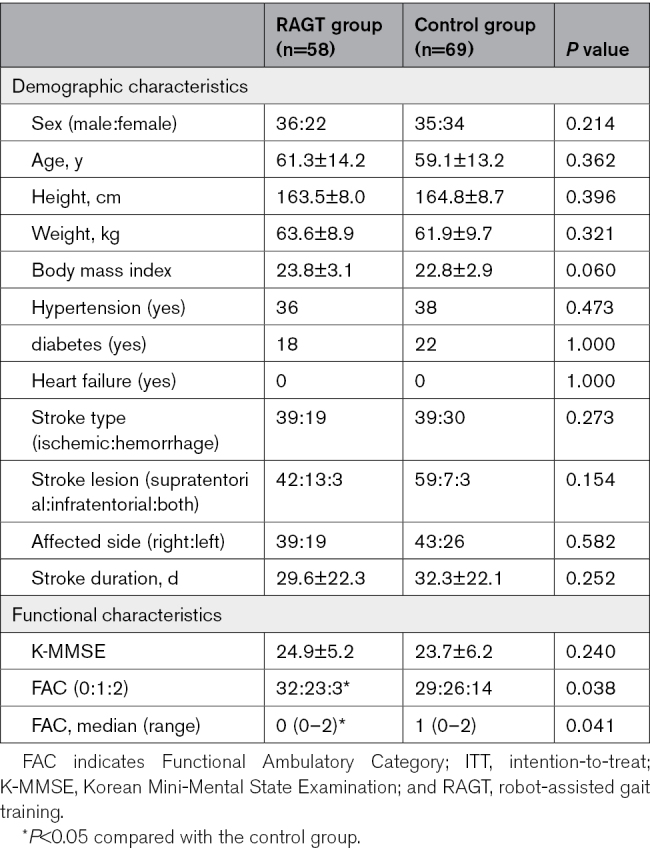
Baseline Characteristics of Participants (ITT Analysis)

### Primary Outcome

**Figure 2.**
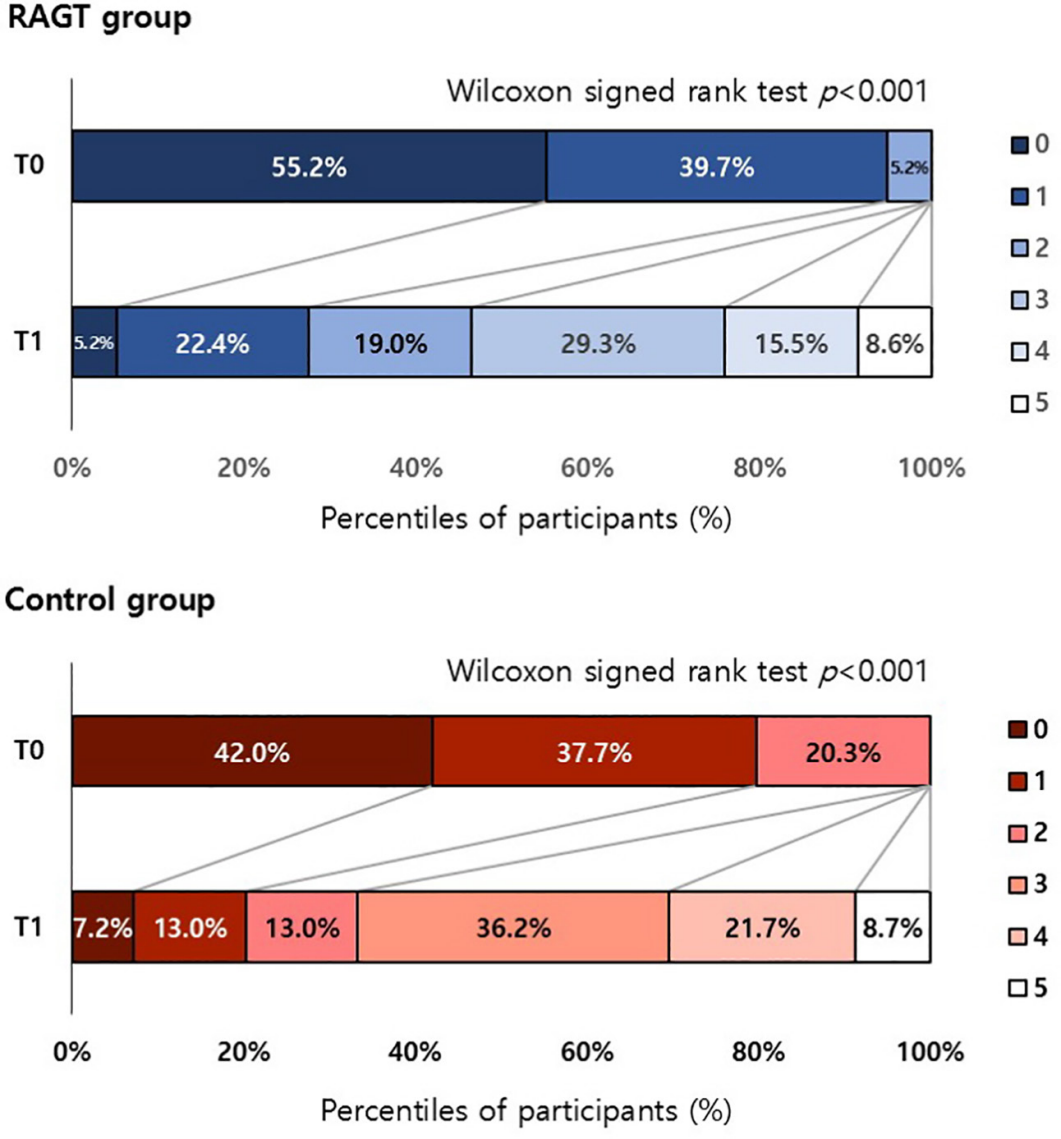
**Improvement of Functional Ambulatory Category (FAC).** Lighter color shades are associated with better ambulatory function in each group. Although both groups demonstrated a significant improvement in FAC from T0 to T1 (*P*<0.05), there was no significant difference in the change of FAC between the 2 groups. RAGT indicates robot-assisted gait training.

Figure 2 illustrates the improvement in FAC among participants in each RAGT and control group. There was a significant improvement in FAC from T0 to T1 in each group (*P*<0.05). The median change (range) of FAC in the RAGT group and the control group was 3.0 (1–5) and 2.5 (1–4), respectively. There was no significant difference in the change of FAC from T0 to T1 between the 2 groups. A rank ANCOVA for the change of FAC from T0 to T1 did not demonstrate a difference between the groups (*F*_[__1125]_=0.729; *P*=0.395).

### Secondary Outcomes

#### Achievement of Independent Gait at T2

**Figure 3.**
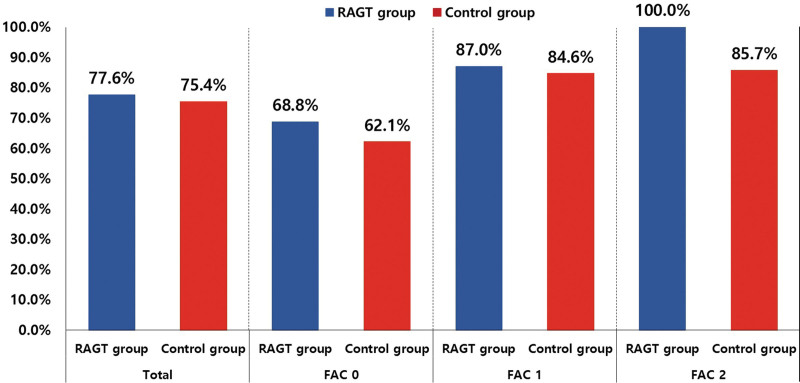
**Achievement rate of participants who walk independently in the robot-assisted gait training (RAGT) and the control group.** According to Functional Ambulation Category (FAC) at T0, there was no significant difference between the 2 groups.

At T2, independent gait was achieved in 77.6% (n=45) of the RAGT group and 75.4% (n=52) of the control group, showing no significant difference between the 2 groups (*P*=0.769). In participants with FAC 0 at T0, 68.8% (n=22) and 62.1% (n=18) of participants demonstrated independent gait at T2 in the RAGT and control groups, respectively. Meanwhile, 87.0% (n=20) and 84.6% (n=22) of participants with FAC 1 at T0 showed independent gait at T2 in the RAGT and control groups, respectively. A subsequent analysis according to the FAC at T0 revealed that there was no significant difference between the 2 groups (Figure 3).

#### Motor and Balance Function

**Figure 4.**
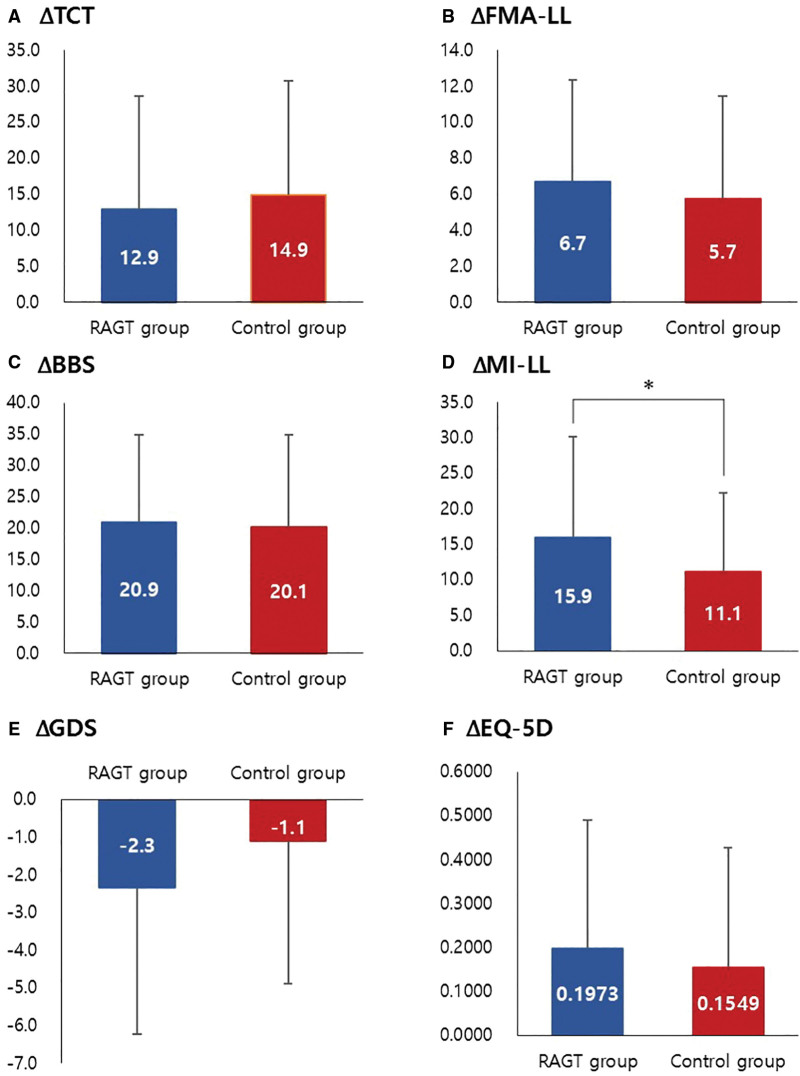
**Changes in behavioral measures after intervention for 4 weeks.** *Y* axis is the change of the value from T0 to T1. The values in each bar represent the mean±SD. BBS indicates Berg Balance Scale; EQ-5D, EuroQol-5D; FMA-LE, Fugl-Meyer Assessment–Lower Extremity; GDS-SF, Geriatric Depression Scale–short form; MI-LL, lower limb score of Motricity Index; RAGT, robot-assisted gait training; and TCT, Trunk Control Test. *P<0.05.

At T0, no significant differences in balance function, as measured by the Trunk Control Test and Berg Balance Score, were observed between the 2 groups. A notable improvement was observed in each group from T0 to T1 (*P*<0.05, Table 2). There was no significant difference in each change of the Trunk Control Test and Berg Balance Score from T0 to T1 between the 2 groups (Figure 4).

**Table 2.**
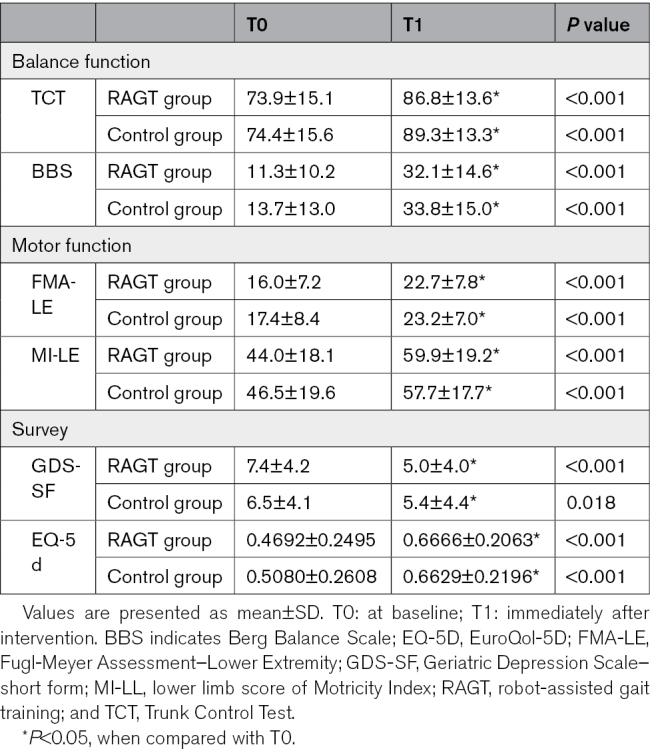
Behavioral Outcome Measures

There was no significant difference in lower extremity motor function, as measured by the Fugl-Meyer Assessment–Lower Extremity and ML-LL, between the 2 groups at T0. A notable improvement was observed in both the Fugl-Meyer Assessment–Lower Extremity and ML-LL scores from T0 to T1 in each group, respectively (*P*<0.05; Table 2). There was no significant difference in the change of Fugl-Meyer Assessment–Lower Extremity from T0 to T1 between the 2 groups. However, the improvement in lower limb score of Motricity Index from T0 to T1 in the RAGT group showed a significantly higher value compared with that in the control group (*P*=0.034; Figure 4).

#### Mood and Quality of Life

There was no significant difference in GDS-SF and EuroQol-5D between the 2 groups at T0. Each GDS-SF and EuroQol-5D showed a significant improvement from T0 to T1 in each group, respectively (*P*<0.05; Table 2). There was no significant difference in each change of GDS-SF and EuroQol-5D from T0 to T1 between the 2 groups. However, the change of GDS-SF scores tended to be higher in the RAGT group compared with the control group without statistical significance (*P*=0.070; Figure 4).

### Adverse Effects

No significant adverse effects were observed in either the RAGT or control groups, including an increase in spasticity, falls, or fractures (Table S1). Table S2 presents a comparison of baseline characteristics between participants who completed the intervention and those who dropped out in the RAGT group. There was no significant difference in all baseline characteristics between participants who completed the intervention and those who dropped out in the RAGT group.

## Discussion

The present study did not reveal that the overground gait training using a torque-assisted exoskeleton plus conventional gait training was more effective than conventional gait training in patients with subacute stroke on the recovery of ambulatory function. However, the overground gait training with an exoskeleton might provide additional lower extremity motor functional improvement in patients with subacute stroke. In addition, there was no significant adverse effect in the overground gait training with an exoskeleton compared with the conventional gait training. These results demonstrated the feasibility and safety of overground gait training using a torque-assisted exoskeleton for 4 weeks in patients with subacute stroke.

The primary objective of measuring changes in ambulatory function before and after 4 weeks of intervention was not achieved in the overground gait training using a torque-assisted exoskeleton compared with conventional gait training. Follow-up assessments up to 3 months postintervention showed no significant differences between groups in the proportion achieving independent walking. These results suggest that 4-week exoskeleton-assisted overground gait training showed no significant difference in comparison to conventional gait training in enhancing ambulatory function in patients with subacute stroke. The outcomes align with previous meta-analyses of treadmill-based robotic gait training,^27^ indicating that overground exoskeleton training may be comparable to conventional rehabilitation for long-term ambulatory improvement in subacute stroke. A major strength is the international, multicenter design across 6 institutions in 2 countries, supporting the generalizability of findings to patients with subacute stroke of various ethnicities in Northeast and Southeast Asia.

The overground gait training using an exoskeleton in this study has been demonstrated to enhance ambulatory function and augment the lower limb score of Motricity Index. The lower limb score of Motricity Index refers to the enhancement of lower limb muscle strength.^21^ Lower limb muscle strength is identified as a pivotal factor in enhancing ambulatory function in patients with subacute stroke.^28,29^ The augmented strength gains observed in the RAGT group relative to the control group in this study may facilitate the further improvement of ambulatory function. The augmented strength gains observed in the RAGT group in this study were likely attributable to the nature of the gait rehabilitation robot. The efficacy of RAGT in enhancing lower limb strength in patients with stroke has been reported in previous studies with tethered gait training robots.^30,31^ In addition, the utilization of an overground exoskeleton for RAGT has been demonstrated to be a efficacious method for enhancing strength.^32,33^ The integration of resistance training into gait training using an overground exoskeleton has been shown to be a productive method for improving strength. The exoskeleton utilized in this study was designed to provide assistance in accordance with the patient’s specific torques, which were automatically detected by a ground contact sensor, encoders in the actuators, and an inertial measurement unit sensor located in a backpack. Consequently, the program was modified to include a resistance exercise component, which required a greater degree of muscular strength.

The rate of attrition was notably high, with the predominant reason being that participants expressed a desire to discontinue their involvement in the study. This finding suggests that the overground gait training with an exoskeleton may present more challenges than conventional gait rehabilitation therapy. Indeed, the RAGT approach entails a substantially greater proportion of walking sessions when compared with conventional rehabilitation within the same timeframe.^4^ A study on treadmill-based robot training, which was conducted on participants similar to those in this study,^34^ reported a dropout rate of ≈18.2%. The predominant causes of attrition in this study and the aforementioned one were fatigue and medical complications. The present study found no significant differences in the characteristics between the dropout and completion groups in the RAGT group. Moreover, no substantial deleterious effects, including musculoskeletal discomfort, were observed in the RAGT group. The factors associated with dropout from robot-assisted gait rehabilitation are presumed to be related to rehabilitation motivation rather than baseline balance or lower limb motor function. To reduce the incidence of participants withdrawing from overground gait training with an exoskeleton, it is recommended that enhanced preregistration training be planned, that more flexible scheduling options be made available, and that active participant support be provided. This may include meticulous observation of discomfort. However, given the absence of an assessment of motivation for rehabilitation in the present study, the validity of these findings remains unconfirmed. Further research is necessary to substantiate these observations and to explore the potential implications for clinical practice.

The study found no specific side effects, such as an increase in spasticity or musculoskeletal pain, were observed in the RAGT group. The overground gait training with a torque-assisted exoskeleton used in this study provides indirect evidence that resistance training in conjunction with functional exercise may prove to be a more efficacious approach. However, it is noteworthy that 4 patients in the RAGT group experienced new major conditions, including stroke recurrence. Subsequent comprehensive clinical analysis revealed that these new major events during intervention were unrelated to the RAGT utilized in this study. Moreover, clinical applications and studies of exoskeletons have been conducted in numerous hospitals to date without safety issues, suggesting that the major side effects observed in this study were likely due to chance. Nevertheless, further elucidation through phase III or IV studies may be warranted.

This study has some limitations. Due to the potential risks associated with the study, patients with severe trunk control impairment were excluded from participation. This aspect could be considered a limitation of the present study. Consequently, based exclusively on the findings of this study, it is not feasible to conclude that exoskeletons can be universally applied to all patients with stroke with impaired ambulatory function. The findings of this study indicate that wearable exoskeletons may demonstrate a higher level of efficacy in patients who maintain relatively good balance function compared with traditional treadmill-based gait training robots. The selection of a suitable gait rehabilitation robot for patients with stroke necessitates a meticulous evaluation of the patient’s particular requirements and functional capabilities. It is essential to match these systems to the characteristics of individual patients.^4,35^ Further research employing a range of gait rehabilitation robots is necessary to better understand the effectiveness of these systems and to improve patient outcomes. This study’s findings are limited to participants in the early subacute stroke phase. Therefore, we cannot definitively conclude that these results apply to patients in all stroke phases. Further research is needed to determine the efficacy of these findings for patients in the late subacute or chronic phase of stroke.

In conclusion, this study demonstrated that overground gait training using a torque-assisted exoskeleton yielded comparable outcomes to conventional gait rehabilitation in improving ambulatory function in patients with subacute stroke, with the augmented gains in lower limb strength. These findings suggest that the overground gait training with a torque-assisted exoskeleton might be a potential intervention for patients with subacute stroke.

## Article Information

### Author Contributions

Dr Chang contributed to conceptualizing the study, acquisition of data, analysis of data, involvement in drafting the manuscript, and final approval of the version to be published. T.W. Kim and H.S. Kim contributed to conceptualizing the study; acquisition of data, and final approval of the version to be published. F.A. Hanapiah, Dr Lee, S.-H. Han, Dr Jia, and D.H. Kim contributed to conceptualizing the study, acquisition of data, and final approval of the version to be published.

D.Y. Kim contributed to conceptualizing the study, acquisition of data, involvement in drafting the manuscript, and final approval of the version to be published

### Sources of Funding

This work was supported by the Korea Medical Device Development Fund grant funded by the Korean government (the Ministry of Science and ICT, the Ministry of Trade, Industry and Energy, the Ministry of Health and Welfare, the Ministry of Food and Drug Safety; Project Number: RS-2021-KD000003).

### Disclosures

All authors report research grants from the Korea Medical Device Development Fund grant funded by the Korean government (the Ministry of Science and ICT, the Ministry of Trade, Industry and Energy, the Ministry of Health & Welfare, the Ministry of Food and Drug Safety (Project Number: RS-2021-KD000003) related to the submitted work.

### Supplemental Material

Tables S1–S2

CONSORT Checklist

## Supplementary Material
