# Supplementary material for: Efficacy of Wearable Exoskeleton for Gait Recovery in Patients With Stroke: A Multicenter Randomized Controlled Trial
Source: Stroke. 2025 Dec 22;57(3):577–86. doi: 10.1161/STROKEAHA.125.052763 (PMC12928783; doi:10.1161/STROKEAHA.125.052763)
Supplement: Supplementary file 1 [file str-57-577-s001.pdf]

Table S1. Changes of affected lower extremity spasticity

		T0	T1
Modified Ashworth scale (0:1:1+:2:3:4)			
Hip extensor	RAGT group	48:7:3:0:0	50:4:3:1:0
	Control group	63:5:1:0:0	52:12:5:0:0
	p-value	0.297	0.202
Knee extensor	RAGT group	49:6:3:0:0	49:7:1:1:0
	Control group	63:5:1:0:0	59:8:2:0:0
	p-value	0.386	0.709
Ankle plantaflexor	RAGT group	47:10:1:0:0	45:6:4:3:0
	Control group	56:11:2:0:0	48:15:6:0:0
	p-value	0.898	0.092

Table S2. Baseline characteristics between participants completed the intervention and dropped out in the robot-assisted gait training group

	Participants completed the intervention (n=58)	Participants dropped out (n=16)	P-value
Demographic characteristics			
Sex (M:F)	36:22	10:6	1.000
Age (yrs)	61.3±14.2	63.9±11.3	0.496
Height (cm)	163.5±8.0	164.6±9.1	0.661
Weight (kg)	63.6±8.9	64.2±9.4	0.787
Body mass index	23.8±3.1	23.7±2.8	0.915
Hypertension (yes)	36	12	0.391
Diabetes mellitus (yes)	18	7	0.380
Heart failure (yes)	0	0	1.000
Stroke type (ischemic:hemorrhage)	19:39	7:9	0.555
Stroke lesion (supratentorial:infratentorial:both)	42:13:3	11:4:1	0.957
Affected side (right:left)	39:19	7:9	0.144
Stroke duration (days)	39.6±23.3	36.6±25.2	0.565
Functional characteristics			
K-MMSE	24.9±5.2	23.4±5.7	0.328
FAC (0:1:2)	32:23:3	7:8:1	0.466
FMA-LE	16.0±7.2	15.7±7.2	0.892
MI-LL	44.0±18.1	43.6±12.3	0.928
TCT	73.9±15.1	68.0±11.2	0.152
BBS	11.3±10.2	14.2±13.4	0.347
GDS-SF	7.4±4.2	8.3±4.0	0.427
EQ-5D	0.4692±0.2495	0.3298±0.3321	0.070

K-MMSE, Korean Mini Mental State Examination; FAC, Functional ambulatory category; TCT, Trunk Control Test; BBS, Berg Balance Scale; FMA-LE, Fugl-Meyer Assessment-Lower Extremity; MI-LL, lower limb score of Motricity Index; GDS-SF, Geriatric Depression Scale-Short Form, EQ-5D, EuroQal-5D-3L
